# Phototoxic or Photoprotective?—Advances and Limitations of Titanium (IV) Oxide in Dermal Formulations—A Review

**DOI:** 10.3390/ijms24098159

**Published:** 2023-05-02

**Authors:** Michał Gackowski, Tomasz Osmałek, Anna Froelich, Filip Otto, Raphaël Schneider, Janina Lulek

**Affiliations:** 1 Chair and Department of Pharmaceutical Technology, Poznan University of Medical Sciences, 6 Grunwaldzka Street, 60-780 Poznań, Poland; mgackowski4@gmail.com (M.G.); tosmalek@ump.edu.pl (T.O.); froelich@ump.edu.pl (A.F.); filip1274@gmail.com (F.O.); 2 CNRS, LRGP, Université de Lorraine, F-54000 Nancy, France; raphael.schneider@univ-lorraine.fr

**Keywords:** titanium (IV) oxide, photoprotection, phototoxicity, dermal formulations

## Abstract

The widespread role of titanium (IV) oxide (TiO_2_) in many industries makes this substance of broad scientific interest. TiO_2_ can act as both a photoprotector and photocatalyst, and the potential for its role in both applications increases when present in nanometer-sized crystals. Its sunlight-scattering properties are used extensively in sunscreens. Furthermore, attempts have been made to incorporate TiO_2_ into dermal formulations of photolabile drugs. However, the propensity to generate reactive oxygen species (ROS) rendering this material potentially cytotoxic limits its role. Therefore, modifications of TiO_2_ nanoparticles (e.g., its polymorphic form, size, shape, and surface modifications) are used in an effort to reduce its photocatalytic effects. This review provides an overview of the potential risks arising from and opportunities presented by the use of TiO_2_ in skin care formulations.

## 1. Introduction

The beneficial effect of sunlight on the functioning of our bodies was already known and described thousands of years ago. Photoexposure in treating rickets and neurological or skin diseases was recommended, for example, in ancient Egypt [1]. Unfortunately, solar radiation also has another, less favorable side. As a form of energy, it can negatively interact with drugs, leading to their decomposition or even their transformation into harmful photoproducts [2]. Photosensitization of active compounds, especially after topical administration, is considered a real-world problem, while their interaction with UV light often leads to serious phototoxic or photoallergic reactions [3]. Literature data evidence the scale of the problem, according to which almost 400 active substances are considered as photolabile [4]. There is a strong need to carefully analyze the photochemical properties of drugs since there is a clear correlation between phototoxicity and skin cancer that has been confirmed [4]. Moreover, treating patients with photodamaged skin is long-term and requires multiple medications, such as antihistamines, steroids, and nonsteroidal anti-inflammatory drugs [5], generating additional costs. One of the possibilities to reduce the effect of light on drugs applied to the skin is the use of excipients that are able to absorb or scatter the radiation, and therefore produce a photoprotective function. To date, one of the most frequently used was titanium (IV) oxide (TiO_2_)—a substance that has received significant press coverage recently [6]. On the basis of scientific analysis, concerning the inclusion of TiO_2_ in various medical products, on 8 September 2021 the European Medicines Agency (EMA) released a document which indicated the need for further research aimed at finding safer alternatives [7]. In short, the main purpose of this report was to exert pressure on the pharmaceutical industry to develop substitutes for TiO_2_ (to date, most commonly used as color and opacifier) within the time-frame of 3 years. This recommendation was adopted in January 2022 and the main focus was on the genotoxic potential of TiO_2_. Although this detrimental effect has been proven for inhaled particles, the current knowledge regarding the use of TiO_2_ on skin surfaces still remains to be clarified and analyzed in detail [8]. The main assumption of the presented review is to assess the current state of knowledge about the advantages (photoprotection) and disadvantages (phototoxicity) of the use of TiO_2_, mainly in dermal formulations. The goal is to answer whether and to what extent a risk may accompany its application to the skin and what are the possibilities of modifying its properties to maintain the photoprotective function while minimizing potentially harmful effects.

## 2. Photolability of Drugs Applied to the Skin

In general, photosensitization can be defined as a process involving the excitation of a given chemical compound by light. Under the right conditions, with a sufficiently high wavelength of light, radiation can lead to photochemical degradation or photochemical transformation into pharmacologically inactive products, which are more likely to cause skin allergies or burns by a phototoxic or photoallergic mechanism [3]. In the case of drugs applied to the skin, such as analgesics [9], antibiotics [10], antiallergics [11], or anti-acne agents [12], UVA radiations are the most harmful. Direct and uncontrolled exposure of the treated skin to sunlight is likely to result in serious side effects and damage that is difficult to recover from [13].

Acute phototoxic reactions often result in processes occurring at the cellular level, such as DNA or cell membrane damage. Typical phototoxicity is accompanied by sudden changes, which, after proper treatment, disappear upon complete recovery. Photoallergic reactions can last significantly longer, since the absorbed energy induces formation of stable haptens with affinity to the cell proteins, followed by production of antigens [3]. It should also be kept in mind that it is not only topically applied drugs that are responsible for photochemical reactions. In the case of substances administered orally, used chronically in long-term therapies, there is a risk of their accumulation in the skin, which can also result in unwanted effects.

**Figure 1:**
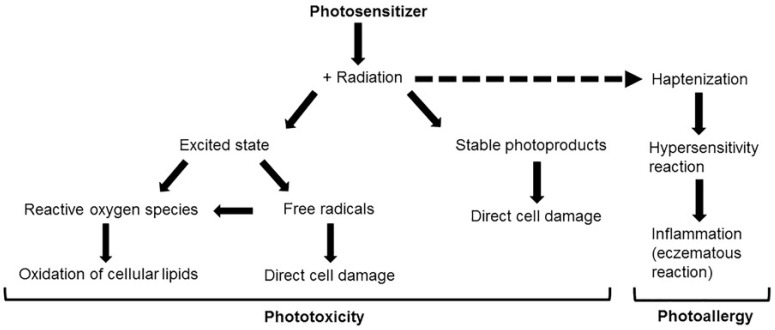
The major pathways of phototoxic and photoallergic tissue damage [4].

The differences between the mechanism of phototoxic and photoallergic reactions are shown in Figure 1, while the characteristics of both types of photosensitization are presented in Table 1. However, reactions caused by topically applied photosensitizers (e.g., in dermatology or ophthalmology) tend to be more severe than those caused by drugs applied systemically [14].

**Figure 2:**
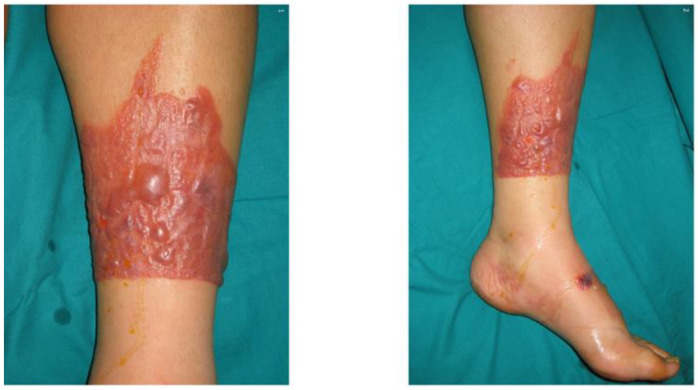
Acute phototoxic reaction following topical application of ketoprofen. Published with the permission of Elsevier [18] published by Elsevier Spain, S.L.U. All rights reserved.

Photoallergy is more relevant to photosensitization than to topically applied agents. Nevertheless, some compounds, such as psoralen and its derivatives, retinoids, and 5-fluorouracil can also cause photosensitization through a phototoxic mechanism [3,14,15]. The severity of the reaction depends on the drug absorption depth, as well as its metabolism in the skin [16]. Drugs in the form of creams or ointments tend to accumulate in the upper layers of the epidermis and primarily cause damage to keratinocytes [17]. However, an acute reaction may be followed not only by necrosis of keratinocytes, but also by epidermal spongiosis, edema, vasodilation, and an infiltration of neutrophils, lymphocytes, and macrophages [14]. An example of an acute photosensitization reaction following the topical application of ketoprofen to sun-exposed skin is shown in Figure 2.

**Table 1:** Characteristics of phototoxicity and photoallergy.

	Phototoxicity	Photoallergy	References
**Clinical symptoms**	erythema, vesicles, and bullae, burning, stinging, hyperpigmentation	eczematous, pruritic rash	[3]
**Histological effects**	necrotic keratinocytes, epidermal degeneration, sparse dermal infiltrate of lymphocytes, macrophages, and neutrophils	spongiotic dermatitis, dermal lymphohistiocytic infiltrate	[3,17]
**Pathophysiology**	direct tissue injury	type iv delayed hypersensitivity response	[2]
**Occurrence after first exposure**	yes	no	[2,3]
**Onset after exposure**	minutes to hours	24 to 48 h	[2,14]
**Dose of agent needed for the reaction**	large	small	[14]
**Cross-reactivity with other agents**	none	common	[2,17]
**Diagnosis**	clinical	photopatch tests	[3,19]

The common active pharmaceutical ingredients recognized as photolabile are presented in Table 2.

## 3. Titanium Dioxide

### 3.1. General Information

**Figure 3:**
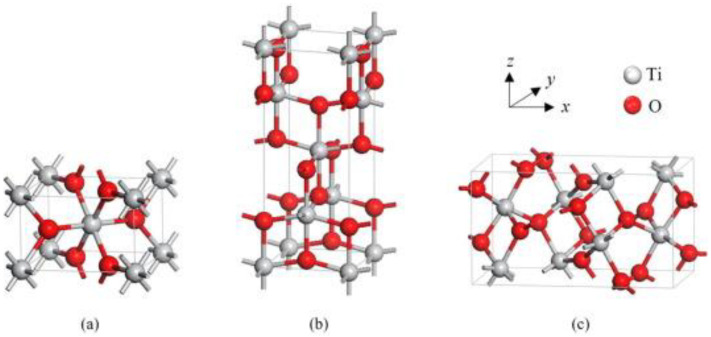
Unit cell structure of TiO_2_ polymorphs rutile (**a**), anatase (**b**), and brookite (**c**). Based on [29].

Crystalline TiO_2_ may exist in three phases, namely rutile, anatase, and brookite (Figure 3) [28].

Variations between these polymorphs involve octahedral units arranged in a three-dimensional space. Importantly, each of the crystal structures exhibits different properties—they can differ in shape, structure, refractive index, and photocatalytic activity [30]. Furthermore, only rutile is stable, while anatase and brookite are metastable and convert to rutile upon heating. The differences between the polymorphs are particularly apparent when comparing their photocatalytic activity. The higher energy gap of anatase and brookite and their superior electron mobility make these polymorphs more reactive than rutile. Moreover, there is a significant difference in the refractive index of rutile and anatase (2.7 and 2.5, respectively). While there are many arguments in favor of the safety of TiO_2_, potential risk factors also need to be considered. On the one hand, excellent photoprotective properties are reported, and on the other hand, the risk of inducing photochemical reactions, as shown in the following sections.

### 3.2. Titanium Dioxide Nanoparticles (NPs) Photoprotection vs. Phototoxicity

TiO_2_ nanoparticles (TiO_2_-NPs) have attracted a considerable attention due to their unique properties, which make them desirable for a wide range of applications. Among industrial applications, they have found a role in cosmetic and pharmaceutical technology. Properties that make it an excellent candidate for the use as a sunscreen include its high photoprotective capacity (strong light scattering) associated with a large photoactive area, insolubility in aqueous media, and transparency. It has been proven that TiO_2_-NPs can act as a shield for both the skin and drugs against UVA and UVB radiations. Moreover, the argument for using TiO_2_-NPs as a sunscreen to date is that they are less photoreactive than other chemical ingredients (especially organic sunscreens), and thus less likely irritating to people with sensitive skin. The EU Scientific Committee on Consumer Safety (SCCS) has approved crystalline TiO_2_-NPs as safe for use in cosmetic products intended for use on healthy, undamaged, or sunburnt skin [31]. As a UV filter, TiO_2_ can be incorporated into cosmetic formulations at a maximum concentration of 25%. However, it has to be considered that the high surface-to-volume ratio makes it legitimate to raise the issue of the increased bio-reactivity of nanomaterials (NMs) and their potential toxicity. Moreover, recent studies and reports on TiO_2_-induced phototoxicity have brought the topic increasingly into the headlines. Therefore, the question of safety must be asked to consider whether chronic exposure to this material is dangerous or not. Although the phototoxic effect of TiO_2_-NPs has already been demonstrated for all polymorphs, it is important to note that different phases can induce a reaction of different severities. However, the main problem with the use of TiO_2_ in products intended for use on the skin surface is its potential to generate ROS [32,33,34,35,36,37,38].

### 3.3. Illuminated TiO_2_-NPs Generate Reactive Oxygen Species (ROS)

Reactive oxygen species are a group of chemically reactive molecules/radicals derived from molecular oxygen. Examples of ROS include superoxide (O_2_^●−^), hydroxyl radical (HO^●^) hydrogen peroxide (H_2_O_2_), and singlet oxygen (^1^O_2_), each of which causes a variety of biological damage and has been implicated in a multitude of diseases, including cancer and ageing [39,40]. As already proven, ROS are deleterious to biological structures (e.g., membranes, lipids, proteins, and nucleic acids) and may be generated by TiO_2_-NPs under certain conditions, e.g., when illuminated with UV light and in an aqueous environment [41].

TiO_2_ is a wide bandgap semiconductor (3.2 eV for anatase) [42] and can only be photo-activated under UV illumination [43]. Only light with a wavelength lower than 387 nm allows for the excitement of an electron from the valence band (VB) to the conduction band (CB). As a result, holes in the VB (h^+^_VB_) and electrons in the CB (e^−^_CB_) are generated and subsequently migrate to the NPs surface to react with water and adsorbed O_2_ molecules, respectively [44]. These reactions are responsible for the production of ROS in the aquatic and aerobic environment. The reactions mentioned are presented as chemical Equations (1)–(6) [44,45].

H_2_O + h^+^ → ^●^OH + H^+^ (1)

O_2_ + 2e^−^ + 2H^+^ → H_2_O_2_ (2)

O_2_^●−^ + O_2_^●−^ +2H^+^ → H_2_O_2_ + O_2_ (3)

H_2_O_2_ + e^−^ → ^●^OH + OH^−^ (4)

H_2_O_2_ + O_2_^●−^ → ^●^OH + OH^−^ + O_2_ (5)

H_2_O_2_ + hv → ^●^OH + ^●^OH (6)

The correlation between TiO_2_-NPs and hydroxyl radical concentrations, UVA light intensity, and concentration of dissolved organic carbon (DOC) with toxicity was assessed by the team of Coral et al. Their study demonstrated that the radical production rate was positively correlated with an increase in TiO_2_-NPs concentration and UVA intensity, and negatively correlated with increased DOC concentrations. Moreover, the authors showed that the concentration of TiO_2_-NPs is a poor predictor of toxicity, when considered alone, and that the concentration of generated hydroxyl radical is a better predictor in assessing the overall risk [45].

An alternative of TiO_2_-NPs phototoxicity was also described. Taking into account the energy transfer of O_2_^●−^ radicals to the holes in the VB, singlet oxygen (^1^O_2_) may also be generated according to Equation (7) [46].

h^+^ + O_2_^●−^ → ^1^O_2_ (7)

^1^O_2_ can oxidize fatty acids or damage nucleotides leading to several oxidative products [46]. The presence of ^1^O_2_ photo-produced by TiO_2_-NPs was confirmed by Fenoglio et al. By modification of the NPs surface, the ROS-generating potential can be decreased. For example, by the introduction to the surface carbonaceous or carbonate/carboxylate-like products, that generation of ROS can be decreased, although the presence of singlet oxygen was confirmed [44].

### 3.4. Factors Influencing the Phototoxic Potential of TiO_2_-NPs

#### 3.4.1. Polymorphic Form

As it was previously mentioned, the polymorphic form of TiO_2_-NPs influences both the material stability as well as the potential of ROS generation. Usually, the rutile phase is less photoreactive than anatase or brookite. This phenomenon is related to a different lifetime of holes (that is longer for rutile) and the electron-trap depth that is shallow for anatase (<0.1 eV), moderate for brookite (~0.4 eV), and high for rutile (~0.9 eV). The deeper the electron trap is located, the less photoreactivity is observed due to lower electron mobility [47]. Another difference between polymorphs is the interaction of these materials with oxygen. Molecular oxygen shows a high affinity to oxygen vacancies. In anatase, the interaction of molecular oxygen with the surface is weaker than for rutile. It explains the difference in the concentration of absorbed oxygen in both forms of TiO_2_-NPs. As shown previously, adsorption of O_2_ relates to an electron transfer from the surface defects, forming superoxide radical anions, which are well known as ROS with a weaker oxidation ability than hydroxyl radicals [48]. The different photoreactivities of anatase and rutile were confirmed in cytotoxicity studies [49].

Tests on immortalized human keratinocytes (HaCaT), both with and without UV light were conducted. In the dark, TiO_2_-NPs (rutile or anatase) did not affect the mitochondrial activity or cell membrane. However, the exposition to rutile NPs may inhibit cell growth and induce OH^−^ generation, which seems to be dependent on the surface properties of nanomaterial, and was confirmed for the hydrophobic surface of rutile NPs. In this study, the cytotoxicity under UVA irradiation was tested and a strong polymorphic-dependent effect was seen. The rutile NPs did not show any cellular effect while the anatase showed strong phototoxicity and induced a marked decrease in mitochondrial activity, cell membrane damage, and the induction of oxidative stress [50]. Another research that settled the higher cytotoxic potential of anatase was conducted by Hering et al. By using both the monolayer cell culture and UV light, the authors confirmed the influence of the polymorphic of TiO_2_-NPs and the material concentration on the cell viability. The results showed that anatase led to a lower effective dose (ED_50_) for all UV doses, the lowest being when irradiated with UVB light. However, rutile showed higher cell viability compared to anatase at the highest intensity of UVB light [51]. The polymorphic-dependent toxicity is mainly connected with the potential to generate ROS, which can damage the cell membrane. This conclusion was also reached by Yin et al. Their work showed that the anatase TiO_2_-NPs are more photoreactive than rutile, being more phototoxic to HaCaT cells [52]. However, some studies confirmed that the mixture of rutile/anatase may be more toxic than smaller pure anatase particles [53,54].

The cytotoxic effect of various polymorphs is also connected with the cellular uptake of NPs. Although the absorption of particles into the cells depends mostly on their diameter, the hydrophilic and hydrophobic surface properties should also be taken under consideration.

#### 3.4.2. Material Size, Surface, and Morphology

In physics—scale matters. The size of the material determines its properties, especially when nanomaterials are considered. The reason that properties of nanomaterials are quite different from bulk concerns both the surface effects and quantum size effects.

The atoms in the surface of the material have fewer neighbors than atoms in bulk, and thus generate a lower coordination and dangling bonds. This makes those atoms less stable compared to bulk atoms. The smaller the particle, the greater the number of surface atoms; therefore, the surface-to-volume ratio scales with the inverse of size. Moreover, in semiconductors (e.g., TiO_2_), the electronic wave functions of conductive electrons are delocalized over the entire particle. Electrons are described as “particles in the box” and the density of state and the energy of particles depend crucially on the size of the box, which leads to a smooth-size dependence. Absorption wavelengths, ionization potentials, and electron affinities are tuned between the atomic values and the work function of the bulk material by varying the size of the NPs. These properties are connected to the availability of electrons for redox reactions. Therefore, the photoreactivity depends on the NPs size [55].

Considering the influence of the size on the cytotoxic effect, tests using the *Sinorhizobium meliloti* bacteria exposed to aqueous dispersions of micrometer-sized TiO_2_ (44 μm) and TiO_2_-NPs (21 nm) under various concentrations were conducted. A hundred percent mortality of the bacteria was reached using TiO_2_-NPs at a concentration of 100 mg/L (under UVA irradiation) and 900 mg/L (under dark conditions). However, the exposure to micrometer-sized TiO_2_ showed that there is no effect on the bacteria in a concentration of less than 300 mg/L (under dark or UVA light irradiation). One hundred percent mortality was only achieved with a nominal concentration of 600 mg/L or more (UVA light irradiated) [56]. This result clearly shows that there is a direct correlation between the effect of material size and the toxic effect.

Sanders et al., tested the uptake and accumulation of TiO_2_-NPs in ARPE-19 cells and showed that after a short-term or long-term exposure, the NPs were observed inside the cells and remained there for an extended period. Moreover, the amount of NPs inside the cells was dose-dependent. However, no clear effects on viability or morphology were observed. The cells that were treated with any of the TiO_2_-NPs and remained in the dark were 100% viable up to the concentration of 30 μg/mL, except for the sample treated with 31 nm-sized anatase/rutile, which showed a dose-related decrease in viability beginning at 10 μg/mL. Moreover, the tests conducted under visible light illumination showed no difference in viability compared to the dark conditions, which is not surprising as TiO_2_ cannot be photo-activated under these irradiation conditions. The differences between samples were observed when cells were exposed to UVA radiation. The outcomes showed clearly that the LC_50_ decreased with the particles size [57]. However, the outcomes of Wyrwoll et al., suggested that these results are only valid on a limited number of particle sizes. The authors showed that NPs of 25 nm were more toxic than those of 10 nm. Although the smaller NPs are able to generate a higher amount of ^●^OH radicals, the sum of all measured ROS (free and surface attached ^●^OH and O_2_^●−^ radicals) was the highest for 25 nm-sized NPs [58]. These phenomena may be explained by the different mechanisms that remain behind the photoactivity. For the particles smaller than 25 nm, the mechanism depends more on optical and electronic properties, including light absorption, scattering efficiencies, and charge-carrier dynamics. In contrast, for particles larger than 25 nm, the photoactivity depends more on the surface area available for redox reactions [59]. Moreover, Wyrwoll et al., confirm that the ionic strength (IS) of the medium highly influences the material toxicity as a high IS promotes material agglomeration and thus sedimentation; therefore, lowering its phototoxicity [58].

Another important factor influencing the toxicity of TiO_2_-NPs is their specific surface area (SSA). As already mentioned, the smaller the particles, the higher the surface/volume ratio. The large surface area of the material allows it to come into close contact with the cells, which is essential for maximizing the damaging effects of ROS, whose lifetime is extremely short. However, particles with the same size may have a different SSA, and therefore produce various photo- and cytotoxicity effects. This effect was tested on RAW264 cells in the absence and presence of light. It was shown that a larger SSA induced higher cyto- and phototoxicity (UV activated) in cells. The increased interactions between the NPs and biomolecules and the greater number of ROS generated contribute to these effects [60].

**Figure 4:**
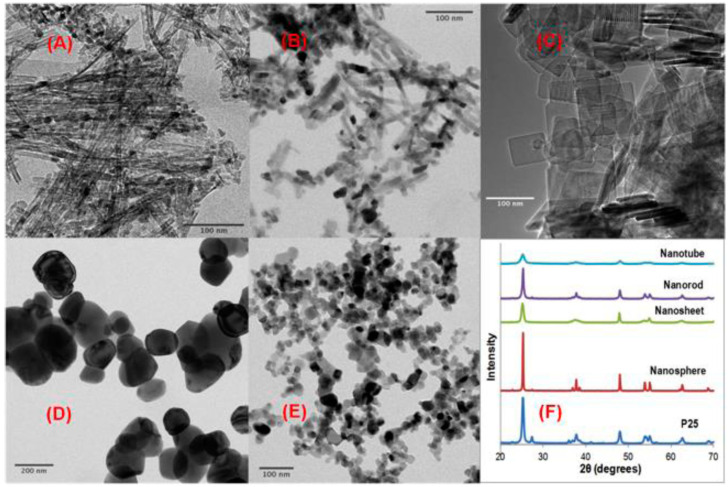
TEM micrographs of TiO_2_ nanotubes (**A**), nanorods (**B**), nanosheets (**C**), nanospheres (**D**), and AEROXIDE^®^ P25 (**E**); (**F**) XRD patterns of different TiO_2_ nanostructures. Reprinted with permission from [61] Copyright 2013 American Chemical Society.

**Figure 5:**
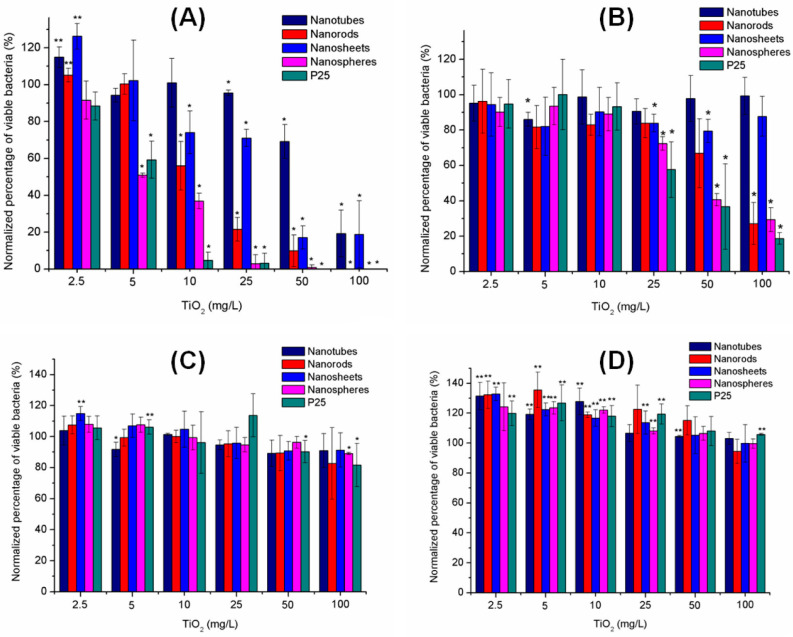
Bacterial viability of *E. coli* and *A. hydrophila* exposed to different TiO_2_-NPs. (**A**,**B**) *E. coli* (**A**) and *A. hydrophila* (**B**) under simulated solar irradiation. The exposure durations for *E. coli* and *A. hydrophila* were 1 h and 20 min, respectively. (**C**,**D**) *E. coli* (**C**) and *A. hydrophila* (**D**) in the dark for 1 h. One asterisk (*) indicates a statistically significant decrease in bacterial viability (*p* < 0.05) compared to the TiO_2_-free control, whereas two asterisks (**) indicate an increase in bacterial viability (*p* < 0.05). Reprinted with permission from [61] Copyright 2013 American Chemical Society.

When considering the physical properties of a material that affect its phototoxic potential, the shape of the particles, which influences their interaction with the cell, should be mentioned. TiO_2_-NPs are usually produced in spherical shapes. However, TiO_2_ may also exhibit low-dimensional nanostructures, such as one-dimensional nanotubes, nanorods, nanobelts, and nanowires or two-dimensional nanosheets. By measuring the membrane integrity of bacteria, the evaluation of how different morphologies influence the cytotoxicity of TiO_2_-NPs was assessed. The outcome showed that the toxic effect strongly depends on the shape with the following order: nanospheres > nanorods > nanosheets ≈ nanorods, and thus indicating that low-dimensional materials are less toxic. Using scanning electron microscopy, it was suggested that the material morphology influences TiO_2_-NPs phototoxicity by governing the alignment of TiO_2_-NPs at the bacterial cell surface. The different shapes that TiO_2_-NPs can take are shown in Figure 4 and the influence of the particles’ shape on bacterial viability is presented in Figure 4 and Figure 5 [61].

The determination of the cytotoxic and phototoxic effects of TiO_2_ cannot be described as a simple function of the photocatalytic reactivity or ROS production. Rather, the evaluation of TiO_2_-NPs phototoxicity must encompass a three-pronged approach, involving the intrinsic photoactivity (influenced by, e.g., polymorphic form, particles size, or specific surface area), aggregation of TiO_2_-NPs (influenced by, e.g., particles size, ionic strength of the medium, or the materials surface charge), and the TiO_2_-NPs/cell interactions (influenced by, e.g., material morphology, specific surface area, or ability to penetrate the cell).

### 3.5. Cytotoxicity as a Response to Photocatalytic Properties of TiO_2_-NPs

The photocatalytic properties of TiO_2_-NPs lead to cytotoxicity, thus inducing apoptosis or, in some cases, necrosis of the cell [62,63]. Necrosis, as premature cell death, can be caused directly by ROS generated by NPs exposed to UV light, which causes damage to the cell membrane. In contrast, apoptosis is a controlled and programmed cell death. In response to cytotoxicity, cells may undergo apoptosis by activating cell signals that cause the cell to break down and die. The mechanism of apoptosis involves a sequence of biochemical events that results in the activation of proteases, such as caspases, that cleave the cell proteins and DNA, leading to cell death. The cell may be induced to initiate apoptosis by an external signal, e.g., in the form of free-oxygen radicals present in its environment [64].

As shown, the type of cell death (i.e., by apoptosis or necrosis) depends on the cell line. Among the mechanisms responsible for cell necrosis, the main one seems to be oxidative stress. By reducing the mitochondrial membrane potential and damaging those structures, the cell becomes devoid of its “engine”. An essential role in the regulation of the mitochondrial membrane potential is played by the mitochondrial permeability transition pore (mPTP)—a non-specific channel between the inner and outer membrane of mitochondria [65]. The disorder in the form of abnormal opening of the mPTP in response to TiO_2_-NPs under UVA irradiation was proven for the HeLa cell line (immortalized epithelial cells), confirming that necrosis plays a key role in cell death. Moreover, the researchers suggest that by regulating the ROS-mPTP pathway, it is possible to influence the cell response to TiO_2_-NPs [66].

However, caspase-dependent apoptosis seems to play a major role in the death of HaCaT cells caused by ROS generated by UV-illuminated TiO_2_-NPs. Exposure of HaCaT cells to those NPs resulted in accumulation in lysosomes via endocytosis or autophagy. Under UVA light, TiO_2_-NPs damage the lysosomal membrane and subsequently stimulate ROS production. Therefore, it was suggested that lysosomal membrane permeabilization (LMP)-dependent oxidative stress plays a critical role in the UVA phototoxicity of TiO_2_-NPs in HaCaT cells [67]. Nonetheless, not only ROS have been proven to induce NPs toxicity. Ren et al., point out that reactive nitrogen species (RNS) also induce apoptotic signaling and pathologies in intracellular redox homeostasis. The authors observed the effects of TiO_2_-NPs on cell glycans—structures that play a regulatory role in various physiological processes, such as sialylation. Sialylation is the glycosylation modification playing a key role in cell adhesion, signaling, and recognition, as well as aging and senescence [68]. Using the HaCaT cells, they have proven the changes in sialic acid concentration in the response of ROS generated by UV-illuminated TiO_2_-NPs, the changes that overall have been reported to cause cancer [69]. Among the biochemical mechanisms that can be influenced by TiO_2_-NPs, Janus kinase/signal transducer and activator of transcription (JAK/STAT) pathway may be disrupted by these NPs. JAK/STAT pathway is known to regulate the development and reproduction in many organisms. In the example of *Caenorhabditis elegans*, the alteration of this pathway was observed, as a response to oxidative stress. Moreover, down-regulation in glutathione activity also appeared. As the JAK/STAT pathway is cross-talked with transforming growth factor beta (TGF-β; the protein that plays an important role in regulating cell growth and extracellular matrix synthesis), the pathway gene expression of this protein was also tested. As with JAK/STAT, significant changes in this gene expression under the influence of TiO_2_-NPs (under UV) were observed, indicating a significant reproductive toxicity [70].

Although the cytotoxicity was proven in cell tests, there is a significant discrepancy between 3D and 2D cultures. In 2D cell culture, NPs are suspended in the cell media. Due to their physicochemical properties, a sedimentation of NPs was observed, as the result of agglomeration, thus increasing the amount of TiO_2_ at the bottom of culture wells. This increases the cellular uptake resulting in an overestimated toxic effect [71]. Moreover, the 2D models do not consider the natural barrier of the skin. Due to the above, many studies have proven that the toxic effects on 2D cell cultures do not reflect in 3D models [50,51].

A review that brought together 50 papers focusing on in vitro and in vivo toxicity testing of TiO_2_ suggests that the genotoxic effect of TiO_2_ cannot be directly confirmed or ignored. The authors point out that more in vitro than in vivo studies should be performed, leading to more positive results of genotoxicity in vitro models than in vivo models. Moreover, they conclude that more well-designed in vivo studies must be performed to assess TiO_2_ genotoxicity [72].

Furthermore, a very recent review including 192 articles with 34 robust datasets does not support a direct DNA damaging mechanism for TiO_2_ in nano- or micro-form [49].

### 3.6. Might Modifications of TiO_2_-NPs Surface Decrease Phototoxicity?

The widespread potential use of TiO_2_ in medicine and cosmetics has led researchers to try to reduce phototoxicity by modifying the particles surface. Most of the modifications involve the introduction of an inert shell on the surface of the NPs. Among the materials used for coating, the most common are silica (SiO_2_), alumina (Al_2_O_3_), zirconium dioxide (ZrO_2_), or siloxanes [36]. However, the main challenge in synthesizing these types of particles with decreased phototoxicity is to suppress the photoactivity with preserved UV-shielding ability. Moreover, due to the potential application of these particles, the shell must be built of inert materials, the role of which is to inhibit the formation of ROS or to scavenge ROS. However, to date, there is no evidence that this method leads to a complete deactivation of the catalytic activity of TiO_2_-NPs [36,73].

One of the most promising materials for shelling TiO_2_-NPs is SiO_2_, mainly due to its wide bandgap of ca. 9 eV. To date, several trials have been reported in which the properties of TiO_2_-NPs have been successfully modified [74,75,76,77]. Notable is the decrease in photocatalytic activity by 85% relative to the TiO_2_ particles while preserving 76% of the original UV-scattering ability [75]. El-Toni et al., decreased the photocatalytic activity of TiO_2_-NPs and the UV-shielding ability was not significantly reduced after coating [78].

Nevertheless, the nanoparticle coating procedure has led to a change in the mechanism of the potential generation of toxic effects by the newly formed material. Based on the model of fish gill cells, Martin et al., point out that the coating influences NPs ability to be taken up by cells. They examined three different materials: TiO_2_/SiO_2_ (core/shell; hydrophilic), TiO_2_/Al(OH)_3_/PDMS (core/shell/shell; hydrophobic), and TiO_2_/Al_2_O_3_/stearic acid (core/shell/shell; hydrophobic). The results showed that particles with hydrophilic surfaces were present inside the cells, whereas those with hydrophobic surfaces were not [79]. Moreover, considering that NPs can be incorporated into the cells raises questions about their safety [80]. Furthermore, Tang et al., showed that NPs coated with silica or alumina exhibited high phototoxic potency that might originate from the interaction of harmful metal ions released from the oxide coating (e.g., Al^3+^) [81]. However, this effect was not confirmed considering the 3D skin model, as the particles could not penetrate the skin barrier.

Reducing the toxicity of TiO_2_-NPs by coating with a chemically inert material carries the risk of other toxicity mechanisms of the resulting core/shell NPs. At the same time, the lack of comprehensive in vitro and in vivo studies of the newly engineered materials suggests that further research is needed to confirm the safety of coated TiO_2_-NPs for medicinal applications.

## 4. Titanium Dioxide Nanoparticles as Photostabilizing Agents in Topical Drug Formulations

The use of TiO_2_ as a photoprotector for photolabile active pharmaceutical ingredients (APIs) in dermal drug formulations is very limited, and to date, was tested only for retinoic acid and ketoprofen. However, all the papers confirm the increased photostability due to the use of this inorganic sunscreen, compared to formulations without TiO_2_ [13,82,83]. Asfour et al., combined all-trans retinoic acid (ATRA) and TiO_2_ for the alleviation of photo-induced sensitivity. The authors confirmed the positive impact of the prepared formulation on product safety in vitro and in vivo studies. They have proven that the addition of 6% (*w*/*w*) TiO_2_ resulted in the enhanced photostability of ATRA by about 2 times, compared to its methanolic solution. Moreover, in vivo studies on mice exposed to direct sunlight for 4 days, confirmed that the prepared formulation alleviated the photosensitization in comparison to the marketed product [82]. The positive effect of TiO_2_ on ketoprofen (KP) photostability has been proven for transdermal patches [13] and gels [83]. By incorporating TiO_2_ into the fabric backing of the patch, the UV transmission through the product was effectively decreased in the UVA region, exhibiting the same increased photostability of KP. Moreover, in vivo studies demonstrated lower photosensitization of the patch with TiO_2_, compared to the KP patch without TiO_2_. In the latter study, the lowered decomposition of KP in the gel was confirmed. The three materials (crystalline TiO_2_, pharmaceutical grade TiO_2_, and TiO_2_ coated with alumina and silica) were tested and the latter-one showed the best effect. The 2 h exposure of the gels with and without coated TiO_2_ to ordinary sunlight caused a decrease in KP concentration from 2.5% to 0.05% (gel without TiO_2_) and from 2.5% to 0.71% (gel with TiO_2_).

Both presented studies conclude the high potential of TiO_2_ in the protection of photolabile API applied to the skin. However, given the scarcity of work on the subject, more in-depth studies and a comprehensive evaluation of the effect of TiO_2_ addition on the formulation stability are required.

## 5. The Use of TiO_2_ in Sunscreens and Cosmetics

The common link between photocarcinogenesis, photoaging, and photosensitivity is their induction by exposure to sunlight. Although sunlight is essential for certain metabolic pathways, such as vitamin D_3_ synthesis, overexposure to UV radiation leads to more harm than benefit [84]. The World Health Organization (WHO) indicates that, as of 2018, there is a reported incidence of approximately 3 million keratinocyte skin cancers and 132,000 melanoma skin cancers, stating that one of every three cancers diagnosed is some form of skin cancer [85]. Given these statistics, it is necessary to provide some protection from UV radiations.

Among the various sun protection strategies, the use of sunscreens is a common process. In general, we can distinguish between organic and inorganic sunscreens. Organic agents (such as, e.g., benzophenone, para-aminobenzoic acid, or menthyl anthranilate) act by absorbing UV radiation, which then leads to the activation of electrons to an excited state. When returning to a stable state, energy is emitted in the form of fluorescent radiation. In contrast, the mechanism of action of inorganic sunscreens (e.g., TiO_2_ and ZnO) exploits the ability of these particles to reflect and scatter UV and visible radiations from a layer of particles [86]. Furthermore, considering the mechanism of action of both types of sunscreens, it is worth noting that inorganic agents show less variability in protective action than organic ones (which absorb UVA and UVB light of narrow wavelength ranges). Furthermore, the structural stability of inorganic sunscreens accounts for their photoprotection even after prolonged UV exposure [87].

As described in the previous paragraph, TiO_2_ exists in different polymorphic forms. For the use as a sunscreen, rutile TiO_2_ is preferred since its absorption starts around 380–400 nm, while for the anatase type, the onset of absorption is located at 360–380 nm. The effectiveness of the photoprotective potential also depends on the shape and size of the particles [88]. Although TiO_2_ can be effective in scattering UV and visible radiations, it does not meet the requirements set by the cosmetic industry when it is micrometer-sized. Its high refractive index (of ca. 2.6) results in a white appearance, which reduces its cosmetic acceptability [89]. For this reason, it is mandatory to reduce the particle size, making the particles transparent, but also shifting the protective spectrum toward shorter wavelengths [90]. The highest visible light scattering efficiency has been proven for particles of around 150–250 nm. Particles with a size of about 10–20 nm are completely lacking in visible light scattering. Therefore, the decrease in particle size to 50–100 nm results in excellent UV absorption and low visible light scattering [88]. However, given the size of these NPs, favored for their aesthetic and physical characteristics, the safety of TiO_2_-NPs has been continuously questioned in many studies. As indicated previously, the cytotoxicity and phototoxicity of NPs have been proven for various cells. Nevertheless, to damage a cell, these particles would have to penetrate the skin, reaching the living cells. However, as some reviews show, inorganic nanoparticles used as sunscreens are safe. Moreover, they are preferable to organic UV filters [91,92]. A comprehensive review presented by Nohynek et al. [93] shows that nanoparticles of TiO_2_ are mostly non-toxic, as they do not penetrate through the stratum corneum and do not affect living cells. Moreover, the authors highlight that in vitro assessment of NPs toxicity does not translate into in vivo toxicity.

For safety reasons, the concentration of TiO_2_ in a cosmetic formulation must not exceed 25%. However, when safety plays a key role, the properties of the prepared formulation, such as rheological profile or API release, are also crucial for the product. Moreover, sunscreens are evaluated in terms of the effectiveness of their protective action expressed as sun protection factor (SPF). The SPF is the ratio of UV energy required to produce minimal erythema on sunscreen-protected skin to the energy required to produce the same erythema on unprotected skin. The higher the SPF value, the more effective the product. Usually, in cosmetics, SPF is between 2 and 50 [94]. Another important factor characterizing sun filters is the critical wavelength (CW). It is the wavelength below which 90% of the area under the absorbance curve resides. The higher the CW, the better sun protection against UVA displaying higher wavelength values. For broad spectrum coverage, the CW must be above 370 nm [95].

Kamel et al., examined how different TiO_2_ concentrations (1%, 2%, 5%, and 7.5%) affected the SPF of the product loaded with rutin. Using in vitro tests, the authors showed that sun protection was 2.04, 2.51, 4.9, and 3.25 times higher (for the TiO_2_ concentrations tested, respectively) than the reference product. The 5% addition of an inorganic filter ensures an SPF of 5.33, which means that more than 80% of UV radiation will be absorbed by the formula. Moreover, the same formula reached the CW of 390.4 nm [94].

Kubác et al., studied three types of TiO_2_-NPs. Using rutile particles with a primary size below 100 nm, they obtained agglomerates with a diameter of 120–150 nm. The authors tested the photoactivity and UV-protective efficacy of rutile TiO_2_, Pretiox^TM^ UVS30W (commercial product; Precheza; rutile deactivated with silica and alumina surface), and Soltex^TM^ TIO/H_2_O (product deactivated with colloidal nanosilica and dialkylsulfosuccinate). The CW of all tested products was about 378 ± 1 nm and decreased by ~1 nm after irradiation. Pretiox^TM^ UVS30W had the lowest photo-acquired potential, showing a non-significant effect on dye stability (test based on Orange I decomposition in the presence of TiO_2_ and UV/visible irradiation), with decomposition rates similar to pure dye. On the other hand, rutile TiO_2_ showed the highest decomposition rate of Orange I, resulting in an undesirable effect on the stability of the other UV filters used in the formulation. The SPF of the tested samples was around 20 for all products and increased slightly after irradiation. Furthermore, none of the TiO_2_ particles tested penetrated the pig skin. More than 70% of TiO_2_ remained on the skin surface, about 20–30% was detected in the stratum corneum/epidermis, and less than 3% in the dermis. As previously reported in other studies, the authors observed no phototoxic potential against the skin. This study showed that, despite the lack of induction of a phototoxic reaction against the skin, the reactivity of NPs may play an important role on the stability of other compounds present in the formulation, and thus the API [88].

Incorporating coated TiO_2_-NPs into sunscreen formulations could be a way to engineer products with better aesthetics and greater safety due to lower ROS production. These conclusions were reached by researchers who coated TiO_2_-NPs with SiO_2_ Al_2_O_3_, ZrO_2_, and sodium polyacrylate (PAANa) using a low-cost and ultrafast sono-chemical strategy. Importantly, all the particles obtained have SPFs similar to those for the original, pure TiO_2_, while having lower photoactivity. Furthermore, TiO_2_/PAANa showed an increase in colloidal stability [96]. The potential of the coating procedure to increase the efficacy of the filters was demonstrated by Bartoszewska et al. [97]. They compared the level of agglomeration and sun protection efficacy of three prepared materials: Pure TiO_2_, TiO_2_/SiO_2_, and TiO_2_/Ag. The best effect was observed for TiO_2_/Ag, as this composite showed the lowest degree of aggregation. Furthermore, TiO_2_/Ag NPs were found to provide the longest sun protection and water resistance, indicating that Ag significantly increased SPF, especially when compared to pristine samples. Furthermore, the SiO_2_ coating was found to be able to dissolve in aqueous environments, leading to an increase in the phototoxicity of the material, which is in line with current knowledge [97,98,99]. Although the coating of TiO_2_-NPs with inorganic materials has shown promising results, attempts have also been made to reduce the photoactivity of NPs with an increase in the sun protection spectrum through the addition of organic particles. Loto et al., coated TiO_2_ with an aqueous extract of native trees from the Chaco region of Argentina, providing the same almost completely reduced photocatalytic activity compared to an aqueous suspension of bare TiO_2_ and high stability in synthetic fresh and seawater. Moreover, the material prepared in this way showed higher broadband photoprotection [100]. Enhanced UV-blocking efficiency of TiO_2_-NPs was also achieved by embedding the particles in cellulose nanofibres (TiO_2_@CNF). The optimized material showed significantly higher UV absorption than pure TiO_2_. Furthermore, TiO_2_@CNF has proven to be considerably less photoactive, as the hybrid degraded only 14.8% of Rhodamine B in a photodegradation test. In comparison, pure TiO_2_ degraded 95% of the dye [101].

## 6. Conclusions

Over the past few years, interest and public awareness of the use of nano-sized TiO_2_ has increased rapidly. This interest is mainly due to the effective sun protection provided by this material [102]. Given the potential risks associated with the photocatalytic activity of TiO_2_-NPs, many studies have been conducted to reduce ROS production. As researches point out, coating the NPs with organic or inorganic compounds seems to be an effective way to achieve this goal. To increase the safety of inorganic sunscreens, particles with reduced photoactivity could be engineered, and thus achieve a higher safety profile due to the minimized interactions between formulation components and skin sensitizers, as well as greater aesthetic value. The addition of TiO_2_ to sunscreens or cosmetics is common. However, the use of this ingredient in topical preparations is underutilized. When case reports confirm the phototoxic or photoallergic potential of drugs applied to the skin, there is a great need to reduce the photolability of photosensitive APIs. Although the number of papers confirming the positive effect of TiO_2_ added to drugs applied to the skin is still small, all confirm a positive effect on formulation stability. Combining the use of an inorganic sunscreen with high photoprotective and low photocatalytic activity with the drug delivery technology seems to be the right way to increase the safety of drug therapy delivered via this route.

There is no doubt that TiO_2_, despite its photoprotective effect, is also phototoxic. Many in vitro studies indicate that, when exposed to UV photons, it induces cell necrosis or apoptosis. The toxic effect of TiO_2_-NPs is confirmed in almost all 2D cytotoxic studies. However, the same effect is not commonly confirmed in 3D studies using real skin models. Extensive review studies on this topic indicate a great need for more toxicity tests that reflect real-world conditions, including naturally occurring skin barriers.

Therefore, when considering the dilemma: TiO_2_ phototoxic or photoprotective, the following should be pointed out:

1. The phototoxic potential of TiO_2_-NPs must always be taken into account when designing cosmetic and drug formulations;
2. Optimization of physical characteristics, such as polymorphic form, size, shape, active surface area, and hydrophilic/hydrophobic profile of the particles makes it possible to reduce the toxicity of the material and to increase the UV/visible light-scattering capacity depending on the intended use of the product;
3. Coating NPs with inert inorganic or organic shells, as well as incorporating them into organic structures, can effectively reduce their toxicity and increase their protective properties;
4. TiO_2_ can be effectively used not only as a sunscreen, but also as a substance enhancing the stability of formulations containing photolabile ingredients;
5. Given the inorganic sunscreen materials available on the market, TiO_2_ appears to be invaluable for effective protection against harmful UV radiations as it has no other equivalent, showing high protection against UVB radiation and moderately high protection against UVA radiation.

Given the studies proving the effectiveness of TiO_2_ in scattering sunlight, the prospect of its use in drug formulations applied to the skin is high, provided that it will not affect the stability of the drug.

## Figures and Tables

**Table 2:** Photosensitivity or photostability assessment for selected pharmacological groups of drugs with the topical route of administration.

Active Compound	Photosensitivity or Photostability Assessment	Conclusions	References
**H_1_-receptor antagonists** (**antihistamines**)			
**Emedastine**	1. pH-dependent photodegradation in the presence of UV/VIS light 2. HPLC-UV analysis—a quantifying percentage of photodegradation 3. UPLC-MS/MS analysis—identifying photodegradation products, their chemical structure, and possible degradation pathways	photolability in the whole range of pH values	[20]
**Epinastine**		photostability at pH 7.0 and 10.0, decreased at 3.0	
**Ketotifen**		moderately photolabile at pH 3.0 and 7.0, completely decreased at 10.0	
**Promethazine**	1. intercalation promethazine into the montmorillonite (mont) matrix (promethazine salt complex) 2. XRD, DSC, and FT-IR—the behavior of complexes during different light exposure times 3. data analysis—obtaining kinetic of photodegradation and drug photostability information	promethazine-mont salt complex demonstrates a higher value of photostability; this compound can develop topical formulation without photosensitization and adverse reactions in the skin	[11]
**Non-steroidal anti-inflammatory drugs** (**NSAIDs**)			
**Ketoprofen**	1. irradiation aqueous ketoprofen solutions 2. LC-MS/MS, HR-MS analysis—identifying photodegradation products 3. spectroscopic analysis—characterization of photophysical properties of photolysis products	ketoprofen is a strongly photolabile drug; it is necessary to further study and determine the behavior of ketoprofen under the influence of sunlight	[21]
**Diclofenac**	1. compounding niosomal gels based on diclofenac and ascorbic acid with antioxidant properties 2. irradiation commercial formulations based on diclofenac and niosomal gels 3. spectroscopic analysis—obtaining kinetic of photodegradation and drug photostability information	photodegradation of diclofenac is oxygen-concentration dependent. Niosomal formulations enhanced diclofenac permeation and strongly increased photostability	[22]
**Naproxen**	1. irradiation aqueous naproxen solutions 2. HPLC-UV analysis—a quantifying percentage of photodegradation 3. toxicity tests—amperometric biosensor based on suspended yeast cell	naproxen and its photodegradation products exhibit toxic properties which lead to yeast cell culture death	[23]
**Retinoids**			
**Isotretinoin**	1. compounding microemulsion based on isotretinoin 2. irradiation isotretinoin-methanol solution and isotretinoin microemulsion 3. spectroscopic analysis—obtaining kinetic of photodegradation and drug photostability information	the inclusion of isotretinoin in the microemulsion matrix increases photostability	[24]
**Tazarotene**	1. irradiation ethanolic solutions containing tazarotene without/in the presence of ZnO, TiO_2_, and benzophenone-derivative UV-filters 2. UPLC-MS/MS analysis—identifying photodegradation products, their chemical structure, and possible degradation pathways 3. MTT—analyzing cytotoxic properties	UV irradiation favors retinoids photodegradation. Photodegradation is UV-filter-dependent that exhibits photoprotective properties	[25]
**Antibiotics and antifungals**			
**Minocycline**	1. development of nanocomposite film based on polyvinyl alcohol and halloysite nanotubes for minocycline delivery 2. XRD, FT-IR, Zeta potential, TG analysis	minocycline is a slightly photolabile drug. polymeric formulations increased photostability	[26]
**Sulfathiazole**	1. irradiation aqueous sulfathiazole solutions 2. LC-MS/MS analysis—identifying photodegradation products 3. antimicrobial assays	irradiated sulfathiazole indicated less antibacterial potency against Escherichia coli	[27]
**Clotrimazole**	1. irradiation clotrimazole-methanol solution with ZnO/TiO_2_ powder-mixture 2. pH-dependent photodegradation in the presence of UV/VIS light 3. UPLC-MS/MS analysis—identifying photodegradation products, their chemical structure, and possible degradation pathways, obtaining kinetic of photodegradation	photodegradation of clotrimazole is strongly pH dependent. Instability is marked at acidic pH	[25]

## Data Availability

Not applicable.
